# A survey of the breast care nurse role in the provision of information and supportive care to Australian women diagnosed with breast cancer

**DOI:** 10.1002/nop2.18

**Published:** 2015-06-02

**Authors:** Tracey Ahern, Anne Gardner, Mary Courtney

**Affiliations:** ^1^ School of Nursing, Midwifery and ParamedicineAustralian Catholic UniversityPO Box 256DicksonAustralian Capital Territory2602Australia; ^2^ School of Nursing, Midwifery and ParamedicineAustralian Catholic UniversityPO Box 456VirginiaQueensland4014Australia

**Keywords:** Health services research, nursing roles, oncology nursing, rural nursing, surveys

## Abstract

**Aim:**

To explore the role of the Australian breast care nurse in the provision of information and support to women with breast cancer, with a focus on the differences experienced depending on geographic work context.

**Design:**

A cross‐sectional study.

**Methods:**

This study conducted in 2013, involved surveying BCNs currently working in Australia, using a newly developed self‐report online survey.

**Results:**

Fifty breast care nurses completed the survey, 40% from major cities, 42% from inner regional Australia and 18% from outer regional, remote and very remote Australia. Patterns of service indicated higher caseloads in urban areas, with fewer kilometres served. Breast care nurses in outer regional, remote and very remote areas were less likely to work in multi‐disciplinary teams and more likely to spend longer consulting with patients. Breast care nurses reported they undertook roles matching the competency standards related to the provision of education, information and support; however, there were barriers to fulfilling competencies including knowledge based limitations, time constraints and servicing large geographical areas.

**Conclusions:**

This was the first Australian study to describe the role of the breast care nurse nationally and the first study to investigate breast care nurses perceived ability to meet a selection of the Australian Specialist Breast Nurse Competency Standards. Important differences were found according to the geographical location of breast care nurses.

## Introduction

Breast cancer is the second most common cancer in the world and the most frequent cancer among women worldwide (Ferlay *et al*. 2013). Consistent with worldwide prevalence, breast cancer is the most common cancer amongst Australian women with incidence continuing to increase (Australian Institute of Health and Welfare (AIHW) 2012). Best practice for care of Australians with breast cancer involves health professionals working collaboratively in multidisciplinary teams aiming to meet the multiple health needs of patients (National Breast Cancer Centre and National Cancer Control Initiative 2003, National Breast Cancer Centre 2005a,b). As a member of the multidisciplinary team, the specialist breast care nurse (BCN) was introduced in Australia in the mid‐1990s to facilitate better continuity of care and psychosocial support (Jones *et al*. 2010) for people undergoing treatment for breast cancer. Specialist breast care nursing has evolved over the past twenty years not only in Australia, but in Canada, Europe, the UK and the US (Eicher *et al*. 2012).

In the Australian context, the BCN role involves assisting people with breast cancer across all stages of the continuum of care, including diagnosis, treatment, rehabilitation and palliative care by providing clinical care, information, education, psychological and emotional support, and taking an active role in the co‐ordination of care (National Breast Cancer Centre 2005b). Further, the BCN model of practice is defined as a specialist practice, therefore requiring a higher level of knowledge and skill in the care of women with breast cancer (National Breast Cancer Centre 2005b). Usual requirements include experience in oncology nursing and the recommended minimum education such as a Bachelor of Nursing and a Post Graduate Diploma of Breast Care Nursing (Yates *et al*. 2007). The scope of specialist breast nursing practice is defined as occurring in the context of a model of care that enables the nurse to work collaboratively with patients contributing to patient‐centred care, while also working collaboratively within the multi‐disciplinary team (National Breast Cancer Centre 2005b).

The consensus view is that BCNs are valued highly by their patients (Eicher *et al*. 2006, Halkett *et al*. 2006, Jones *et al*. 2010, Reed *et al*. 2010). However, despite advances in healthcare initiatives including the introduction of BCNs, women with breast cancer still have high unmet support needs. Several studies have found that many women do not have adequate information about their disease and treatment, nor receive enough practical and emotional support from health professionals (McGrath *et al*. 1999, Girgis *et al*. 2000, Raupach & Hiller 2002, Davis *et al*. 2004, Vivar & McQueen 2005, Aranda *et al*. 2006, Lawler *et al*. 2011). Historically, addressing these needs for rural or remote Australian women has proven more difficult because cancer service capabilities in Australia are restricted by many factors including challenges in attracting and retaining a sufficiently skilled workforce and servicing large geographical areas (National Breast Cancer Centre 2001, Health Workforce Australia 2013). For example, in Australia, the large geographical mass means that a women living in the rural town of Richmond, Queensland, would need to travel 500 km to reach the closest regional hospital offering cancer treatment services. Further, to have their treatment in the closest metropolitan city, they would need to travel 1584 km to Brisbane, Queensland.

### Background

In Australia, BCNs are either employed by the McGrath Foundation or by public or private healthcare providers. In 2013, at the time of this study, there were no existing statistics on BCNs working in Australia; however, it was estimated that there were around 400, with 84 of these being employed by the McGrath Foundation (McGrath Foundation 2013, Paynter 2013).

Like many nurses who work in advanced practice roles or specialized practice areas, Australian BCNs practice in a variety settings (Yates *et al*. 2007), with a range of expectations for the role, leading to lack of role clarity (Eicher *et al*. 2006, Lowe *et al*. 2011). Previous research shows there is some doubt about whether all Australian women with breast cancer have adequate access to a BCN (Campbell *et al*. 2006, Eley & Rogers‐Clark 2012). Furthermore, there has been debate about the smaller caseload of BCNs in rural and remote areas, suggesting the role of the BCN in these settings is unsustainable (National Breast Cancer Centre and National Cancer Control Initiative 2003).

The research literature about Australian BCNs consists of predominantly exploratory, single site studies conducted primarily in metropolitan areas (Ahern & Gardner 2015) indicating a need for further research to investigate differing BCN roles in varying geographical and practice contexts. This study was designed in response to this finding and, therefore, explores the role of the Australian BCN in providing information and support to women diagnosed with breast cancer, with a particular focus on reporting the differences experienced in varying geographic work contexts. The research questions addressed are:

1. What role differences occur between rural/remote and urban BCNs in Australia?
2. How does the self‐report role of an Australian BCN compare with Australian Specialist Breast Care Nurse Competency Standards relating to the provision of education, information and support?

## The study

An online survey, known as the Breast Care Nurse Survey was developed for this study and issued using a cross‐sectional design. Ethical approval was granted by the Australian Catholic University Human Research Ethics Committee (approval number 2013 196N). Participants were BCNs currently working in Australia recruited using snowball sampling. Of the 60 participants who began the survey, 50 completed it, giving a completion rate of 83%. Consent was indicated by submission of a completed survey and this was explained in the participant information.

**Figure 1:**
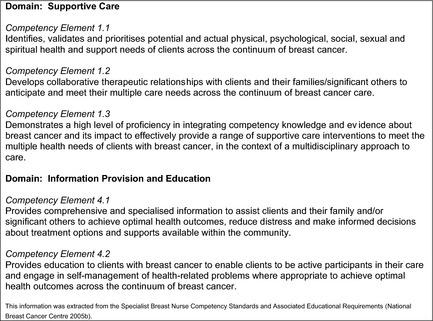
Specialist breast nurse competency standards used to guide Section Three of the Breast Care Nurse Survey.

A literature search revealed no suitable pre‐existing survey instrument. Therefore, a new survey instrument was developed, consisting of 59 structured items and allowance for 26 open‐ended responses, divided into three sections. Section 1 (13 items) collected demographic information about participants. Section 2 (20 items) collected primarily interval and ratio data about caseloads, consults and multi‐disciplinary team involvement. Section 3 (26 structured items with Likert scales and allowance for additional free text) was based on the Specialist Breast Nurse Competency Standards. In this section, the performance criteria for Specialist Breast Nurse Competency Standards 1·1, 1·2, 1·3, 4·1 and 4·2 (see Figure 1) were used as a framework for generating items to explore the breadth of the BCN role in the provision of education, information and support, and the perceived barriers to undertaking each of the performance criteria. Details of development, face validity and reliability of the survey are being prepared for publication separately.

All data were stored electronically in password protected files. Detailed address data were collected to identify the geographic location of each participant; however, names of participants were not collected. Collection of Internet Protocol (IP) address of each participant was a default process of the online survey software. However, steps were taken to ensure that confidentiality and privacy were maintained by first deleting all IP addresses from the database and identifying each record by a unique number. Second, an Australian Bureau of Statistics (ABS) Remoteness Area (RA) code was manually allocated to the physical residential/workplace address of each participant to identify their geographical location (Australian Bureau of Statistics, 2013a). These RA codes were consistent with the latest available information from the ABS and were allocated using an address coding tool on the ABS website (Australian Bureau of Statistics, 2013b). Once the RA code was allocated, all other information relating to place of residence was deleted from the database to protect the identity of participants.

Structured items collected both nominal and ordinal data and were analysed using descriptive statistics with SPSS Statistics software, version 20·0 (IBM Corp 2011). Categories where cell numbers were small were combined. For example, number of years working as a BCN was collapsed from five categories down to three. Thematic analysis was applied to open‐ended questions, where responses were used to add richness to the quantitative data.

## Results

**Figure 2:**
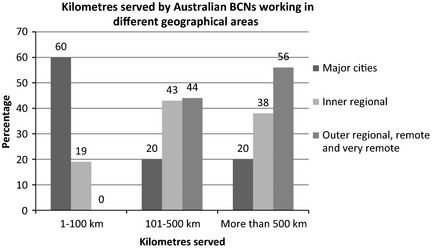
Kilometres served by Australian BCNs working in different geographical areas.

Fifty BCNs completed the survey. Respondents working in major Australian cities comprised 40% (*n* = 20) of the total sample. Forty‐two per cent (*n* = 21) worked in inner regional Australia, with the remaining 18% (*n* = 9) working in outer regional, remote or very remote Australia. Respondents worked in a variety of healthcare settings. Figure 2 shows kilometres served by BCNs working in different geographic areas.

### Breast care nurse education, experience and qualifications

The majority of respondents had worked fewer than 6 years as a BCN (64%, *n* = 32) and this pattern was consistent regardless of geographic location (Table 1). Forty per cent of respondents were employed full time. Most BCNs employed in major cities worked between 3 and 5 days per week (80%, *n* = 16) in contrast to less than half of those employed in outer regional, remote or very remote areas. Overall, BCNs in outer regional, remote and very remote areas also reported being less highly qualified.

**Table 1:** Breast care nurse education, experience and qualifications

Characteristic	Total *n* = 50		Major Cities *n* = 20		Inner Regional *n* = 21		Outer regional, remote or very remote *n* = 9	
	*n*	%	*n*	%	*n*	%	*n*	%
Years working as BCN								
0‐5 years	32	64	13	65	12	57	7	78
6‐10 years	11	22	2	10	7	33	2	22
More than 10 years	7	14	5	25	2	10	0	0
Employment basis								
Full time	20	40	8	40	9	43	3	33
Part time	30	60	12	60	12	57	6	67
Percentage of work week as BCN								
0·5‐2·5 days per week	16	32	4	20	7	33	5	56
3‐5 days per week	34	68	16	80	14	67	4	44
Highest Qualification								
Hospital Trained, Bachelor of Nursing	5	10	2	10	0	0	3	33
Graduate Certificate, Graduate Diploma, Master of Nursing, PhD	45	90	18	90	21	100	6	67
Hold BCN Qualification^a^								
Yes	42	84	17	85	18	86	7	78
No	8	16	3	15	3	14	2	22

a Not all of these qualifications are tertiary level.

### Current patient and consult characteristics

Higher caseloads and higher percentages of patients newly diagnosed with breast cancer were seen in major cities and inner regional areas (Table 2). Results indicate that BCNs in outer regional, remote and very remote areas were more likely to continue to follow patients from diagnosis through to and after completion of adjuvant treatment, than BCNs in major cities. Ninety percent of all BCNs reported seeing patients within one week of diagnosis, with 64% reporting consulting patients at least weekly within the first month of diagnosis. No BCNs reported spending less than 20 minutes each consult with breast cancer patients and BCNs in outer regional, remote and very remote areas were more likely to spend an hour or more consulting with patients.

**Table 2:** Current patient and consult characteristics

Characteristic	Total *n* = 50		Major cities *n* = 20		Inner regional *n* = 21		Outer regional, remote and very remote *n* = 9	
	*n*	%	*n*	%	*n*	%	*n*	%
Case load								
0‐50 patients	26	52	11	55	8	38	7	78
51‐100 patients	12	24	3	15	8	38	1	11
More than 100 patients	12	24	6	30	5	24	1	11
Newly diagnosed breast cancer patients per month								
1‐10 patients	35	70	10	50	16	76	9	100
11‐20 patients	11	22	6	30	5	24	0	0
More than 20 patients	4	8	4	20	0	0	0	0
When patients are seen								
At diagnosis	44	88	18	90	18	86	8	89
Pre‐operatively	45	90	16	80	20	95	9	100
Post‐operatively	46	92	17	85	20	95	9	100
During adjuvant chemotherapy/radiotherapy	43	86	15	75	19	90	9	100
After completion of adjuvant treatment	43	86	14	70	20	95	9	100
How soon consults occur after diagnosis								
At diagnosis	14	28	9	45	3	14	2	22
Within one or two days	14	28	3	15	9	43	2	22
Within one week	17	34	6	30	6	29	5	56
More than a week	5	10	2	10	3	14	0	0
Regularity of consults within the first month of diagnosis								
Never	2	4	2	10	0	0	0	0
Daily	2	4	1	5	1	5	0	0
At least once per week	32	64	11	55	14	67	7	78
Once a fortnight	2	4	1	5	1	5	0	0
Once per month	1	2	0	0	1	5	0	0
As needed	17	34	8	40	5	24	4	44
Time allocated to a consult								
Up to 20 minutes	0	0	0	0	0	0	0	0
21‐30 minutes	21	42	13	65	7	33	1	11
Up to 1 hour	25	50	6	30	12	57	7	78
More than one hour	4	8	1	5	2	10	1	11

### Consult methods and multi‐disciplinary team involvement

Regardless of geographic location, BCNs mostly used face‐to‐face and telephone consultations, only spending 10% of their working week using electronic consultations. Those working in geographically distant areas report less likelihood of involvement in multidisciplinary teams (MDTs). Overall, the majority of BCNs reported spending up to 30% of their working week in MDT meetings and most of these reported feeling encouraged to share their views in MDT meetings (Table 3).

**Table 3:** Consult methods and multi‐disciplinary team involvement

Characteristic Measured	Total *n* = 50		Major cities *n* = 20		Inner regional *n* = 21		Outer regional, remote and very remote *n* = 9	
	*n*	%	*n*	%	*n*	%	*n*	%
Face‐to‐face consults (*n* = 50)								
Up to 50%	31	62	7	35	16	76	8	89
More than 50%	19	38	13	65	5	24	1	11
Telephone consults (*n* = 50)								
Up to 50%	33	66	17	85	12	57	4	33
More than 50%	17	34	3	15	9	43	5	56
Electronic communication consults (*n* = 50)								
Up to 10%	47	94	19	95	21	100	7	77
More than 10%	3	6	1	5	0	0	2	22
Do you work in a MDT? (*n* = 50)								
Yes	44	88	19	95	19	91	6	67
No	6	12	1	5	1	9	3	33
If yes								
What percentage of your week is spent in MDTMs? (*n* = 44)								
Up to 30%	32	73	11	58	17	89	4	67
31‐60%	9	20	6	32	1	5	2	33
More than 60%	3	7	2	11	1	5	0	0
Are you encouraged to share your view in MDTs about patient care? (*n* = 43)								
Yes, always	23	53	11	58	10	56	2	33
Yes, but only if I have been working with the patient	10	23	5	26	3	17	2	33
No, but I feel free to share my view	10	23	3	16	5	28	2	33

### Perceived ability to undertaking competencies related to provision of information and support

Respondents reported regularly undertaking the performance criteria embedded in competency standards 1·1, 1·2, 1·3, 4·1 and 4·2 (National Breast Cancer Centre 2005b). Analysis of relationships using cross tabulation between responses and years' experience revealed those with more than 5 years' experience were more likely to reporting to ‘always’ undertake performance criteria in 22 out of the 26 criteria listed. Relationships between responses and level of education revealed those with post‐graduate education were more likely to report ‘always’ undertaking performance criteria in 19 out of the 26 criteria listed. Notably, much lower percentages of BCNs perceived an ability to ‘always’ undertake performance criteria 1·1c, than was reported in any of the other performance criteria listed and these percentages were equivalent regardless of levels of experience and education.

### Barriers to undertaking competencies related to provision of information and support

Free text information revealed the most common barriers to undertaking performance criteria were large patient loads, limited time constraints and knowledge base limitations. In the supportive care domain, BCNs working in rural or remote centres reported limited resources and limited access to other healthcare workers and identified geographical distances to main treatment centres as barriers to fulfilling competencies. The existence of ethnic and cultural barriers was also acknowledged, especially by those working in remote and very remote settings.

In the domain of information provision and education, a number of BCNs reported lack of private spaces to address matters needing discretion. Some reported they lacked experience, knowledge, skills and confidence dealing with sensitive matters, but many explained they were increasing their skill and knowledge base through education. Those in geographically isolated areas reported that although ongoing education was available, access was difficult due to factors such as distance, cost and time away from work. Free text responses also revealed the difficulties experienced accessing the MDT due to geographical distance. Additionally, some BCNs reported MDT meetings were held infrequently, leading to barriers in provision of timely advice.

## Discussion

Current evidence about the role of the Australian BCN providing information in support to women with breast cancer is lacking (Ahern & Gardner 2015). This study provides insights into the role of the Australian breast care nurse particularly in the context of different geographical locations.

### A comparison of BCNs based on geographical location

As the majority of respondents had less than 6 years' experience in their position, assessing the educational needs of this group and providing additional educational support may be required. Access to additional education through continued professional development (CPD) is an important factor regardless of geographical location and ensures that nursing practice is evidence‐based, maintains best‐practice standards and meets the current needs of communities (Black & Farmer 2013).

Although the ratios of full‐time to part‐time BCNs were consistent across the three geographical areas, there were major differences in total working hours. Perhaps, like many other rural nursing roles, BCNs working in outer regional, remote and very remote areas may be predominantly part‐time in this role and combine the BCN role with other nursing roles, increasing role diversity (Francis & Mills 2011).

The higher caseloads experienced by those working in major cities and inner regional areas may contribute to reduced regularity and duration of consult time. These results support findings of Jones *et al*. (2010) who noted BCNs in metropolitan areas had a wider range of responsibilities, higher caseloads with the potential for more limited care being provided to patients.

Findings related to length of consultations and regularity with which BCNs see their patients throughout the cancer trajectory suggest that women in outer regional, remote and very remote areas may have higher levels of access to their BCNs and therefore more time and opportunity to receive information and support from their BCN. This reinforces the findings from a previous study that found a statistically significantly higher percentage of women in outer regional, remote and very remote areas reported using the BCN for support than women in major cities and inner regional areas (Ahern *et al*. 2014). An increase in the number of BCNs available to meet the needs of women in major cities and inner regional areas would decrease caseloads, enabling BCNs in these areas to increase regularity and duration of consultations, potentially improving the levels of BCN support experienced by these women.

Limited use of electronic consultations were surprising considering that Internet‐based telemedicine is readily available and regularly used by many health professionals (Fatehi *et al*. 2014). Increased uptake of the telemedicine model would provide advantages for BCNs and patients alike saving BCN travel time, while allowing patients the convenience of consulting from the privacy of their own home (Sabesan & Piliouras 2009).

### BCN perceived ability to undertake competencies consistent with their role

Perceptions of BCNs with more experience and/or higher qualifications associated with higher levels of ability to undertake competencies related to the provision of information and support reinforce once again the benefit of continued professional development, training or education relevant to BCN roles. Importantly, BCN perceptions highlighted a specific area of need to enable them to more confidently meet performance criteria 1·1c. This performance criterion indicates that BCNs are required to routinely assess all clients for psychosocial risk factors and distress at the time of diagnosis and on a regular basis using a systematic evidence‐based approach (National Breast Cancer Centre 2005b). Therefore, BCNs may need further education and access to appropriate evidence‐based screening tools to assist them to become more confident and competent in addressing psychosocial and psychological needs of patients.

Online courses are one way of delivering CPD to rural and remote nurses through organizations such as CRANAplus and EdCaN (Australian Government 2010, CRANAplus Incorporated 2014). Each offer web‐based learning resources useful to rural nurses and within cancer control, but more specific resources for BCNs could be developed. However, through an open forum discussion at a national cancer nurses conference, the point was made that often BCNs fail to take up online education that is offered (Ahern 2014), although the reason behind the lack of uptake are unknown.

Reported barriers to undertaking the selected competencies included a lack of culturally appropriate resources available to meet the multiple care needs of women with breast cancer. While there are now resources available through the Breast Cancer Network Australia and Cancer Australia, at the time this study was conducted these resources may not have been readily available (Cancer Australia 2012, Breast Cancer Network Australia 2013).

### Policy and research implications

Continued professional development (CPD), training and support are recommended for all BCNs (Yates *et al*. 2007). It is well documented that continued professional development and education of nurses in the rural setting is difficult due to limited resources (Fahey & Monoghan 2005, McCoy 2009, Health Workforce Australia 2013); however, these are critical elements supporting the role and professional practice of BCNs (Francis & Mills 2011, Black & Farmer 2013). Therefore, individual BCNs should commit to CPD offered and employers need to support and fund nurses to undertake CPD through post‐graduate study and clinical learning opportunities (Black & Farmer 2013). Ongoing assessment of gaps in the education and training provided to BCNs is recommended for educating bodies, and additionally, the reasons for failure to take up online CPD is an area worthy of further investigation.

The lack of access to and participation for BCNs in outer regional, remote and very remote areas to multi‐disciplinary teams compared with their peers in major cities and inner regional areas is consistent with a recent study evaluating the McGrath BCN service which found 47% of rural BCNs participated in MDT meetings (Paynter *et al*. 2013). Multidisciplinary care benefits both clinicians and patients and it is well recognized that cancer care is most effectively delivered by MDTs (Catt *et al*. 2005, National Breast Cancer Centre 2005a, Health Workforce Australia 2013). The BCN is positioned between cancer specialists and patients enabling the nurse to advocate for patients and assist in their medical management (Amir *et al*. 2004), thus the importance of BCN involvement in MDT meetings conducted by the cancer treatment team. Diverse geographical locations make it difficult for face‐to‐face MDT meetings. However, Internet and videoconferencing software can be used not only for outpatient consultations, but also case discussions between health professionals (Doolittle & Spaulding 2006, Sabesan *et al*. 2012). Therefore, it is recommended that clinicians seek opportunities to use videoconferencing technologies enabling inclusion of BCNs from all geographical areas in regular MDT meetings. Current availability of and use of videoconferencing technologies by MDTs is an area in need of more research.

### Strengths and limitations

Importantly, this is the first study to describe the Australian BCN role nationally and to investigate BCNs' perceived ability to meet the Specialist Breast Nurse Competency Standards relating to the provision of education, information and support (National Breast Cancer Centre 2005b). Limitations include using a newly developed self‐report survey tool and a small convenience sample. Further validation of the instrument would be beneficial before it is used in other contexts.

## Conclusion

This study reports the differences in characteristics and practice of BCNs working in urban areas and those working in regional, rural and remote areas. Findings suggest BCNs in outer regional, remote and very remote areas require improved support through better access to continued professional development and training, and better inclusion in MDT meetings. Innovative use of web‐based resources such as online training and videoconferencing should be investigated and used to achieve improved access to CPD and MDTs for BCNs working in outer regional, remote and very remote areas. For BCNs working in major cities and inner regional areas, an increase in numbers of employed BCNs may decrease caseloads and increase patient contact time. All recommendations have the capacity to improve the professional practice of Australian BCNs and outcomes for women with breast cancer.

## Conflict of interest

There are no conflicts of interest declared.

## Author contributions

All authors have agreed on the final version and meet at least one of the following criteria [recommended by the ICMJE (http://www.icmje.org/recommendations/)]:

- substantial contributions to conception and design, acquisition of data, or analysis and interpretation of data;
- drafting the article or revising it critically for important intellectual content.
